# Differential antiviral immunity to Japanese encephalitis virus in developing cortical organoids

**DOI:** 10.1038/s41419-018-0763-y

**Published:** 2018-06-18

**Authors:** Boya Zhang, Yangzhige He, Yanpeng Xu, Fan Mo, Tingwei Mi, Qing Sunny Shen, Chunfeng Li, Yali Li, Jing Liu, Yihui Wu, Guilai Chen, Wenliang Zhu, Chengfeng Qin, Baoyang Hu, Guomin Zhou

**Affiliations:** 1 Department of Anatomy, Histology and Embryology, School of Basic Medical Sciences, Shanghai Medical College, Fudan University, Shanghai, 200032 PR China; 2 Key Laboratory of Medical Imaging Computing and Computer Assisted Intervention of Shanghai, Shanghai, 200032 PR China; 3 State Key Laboratory of Stem Cell and Reproductive Biology, Institute of Zoology, Chinese Academy of Sciences, Beijing, 100101 PR China; 4 School of Life Sciences, Tsinghua University, Beijing, 100084 PR China; 5 State Key Laboratory of Pathogen and Biosecurity, Beijing Institute of Microbiology and Epidemiology, Beijing, 100071 PR China; 6 Beijing Key Laboratory of Cardiometabolic Molecular Medicine, Institute of Molecular Medicine, Peking University, Beijing, 100871 PR China

## Abstract

Japanese encephalitis (JE) caused by Japanese encephalitis virus (JEV) poses a serious threat to the world’s public health yet without a cure. Certain JEV-infected neural cells express a subset of previously identified intrinsic antiviral interferon stimulated genes (ISGs), indicating brain cells retain autonomous antiviral immunity. However, whether this happens in composited brain remains unclear. Human pluripotent stem cell (hPSC)-derived organoids can model disorders caused by human endemic pathogens such as Zika virus, which may potentially address this question and facilitate the discovery of a cure for JE. We thus generated telencephalon organoid and infected them with JEV. We found JEV infection caused significant decline of cell proliferation and increase of cell death in brain organoid, resulting in smaller organoid spheres. JEV tended to infect astrocytes and neural progenitors, especially the population representing outer radial glial cells (oRGCs) of developing human brain. In addition, we revealed variable antiviral immunity in brain organoids of different stages of culture. In organoids of longer culture (older than 8 weeks), but not of early ones (less than 4 weeks), JEV infection caused typical activation of interferon signaling pathway. Preferential infection of oRGCs and differential antiviral response at various stages might explain the much more severe outcomes of JEV infection in the younger, which also provide clues to develop effective therapeutics of such diseases.

## Introduction

Japanese encephalitis (JE) caused by Japanese encephalitis virus (JEV) is one of the most common viral inflammation diseases, particularly in wide area of Asia. In endemic countries, JE occurs primarily among children aged less than 10 years. JEV infection induces non-cell necrotic plaques accompanied by nodules of glia, edema, bleeding, and inflammatory infiltration in multiple brain regions, and usually cause serious neurologic sequelae including the childhood morbidity and mortality^1–5^. Although JE vaccine significantly controls the spread of JE, no effective cure is available for the JEV-infected patients. JE remains one of the most serious threats to public health^6^.

During JEV infection, proinflammatory cytokines and chemokines concertedly trigger neuronal damages. In vitro assays indicate that JEV preferentially infects neural precursor cells and glial cells, rather than neurons^7^. Activated microglia and astrocyte secrete chemotactic cytokines, which attract the inflammatory cells^8^. Innate immune response plays an important role in defensing against viral infection as well participates in the inflammatory response^9^. Upon viral infection, pattern recognition receptors (PRR) recognize the pathogen-associated molecular patterns (PAMPs) and then activates the expression of interferons (IFNs), which then bind to receptors on nearby cells and induce the expression of waterfall of antiviral interferon stimulated genes (ISGs)^10–12^.

Unlike most cells, pluripotent embryonic stem cells (ESCs) do not produce type I IFNs in response to viral infection, and they respond weakly to exogenous IFNs^13, 14^. Upon differentiation, neural stem cells, as well as progenitors at various stages of differentiation express a subset of genes previously classified as intrinsic ISGs for antiviral protection, indicating differentiating and differentiated cells retain autonomous antiviral immunity^15^. However, in the developing brain, how the immune response is activated upon viral infection, and how the infection and immune response affect the cortical neurogenesis remains unknown.

Lately, hPSC-derived three-dimensional (3D) organoids can mimic developing organs such as brain^16^, retina^17^, and pituitary gland^18^. In particular, organoids of entire brain^19, 20^ and brain-region-specific organoids^21^ can model specific human brain infectious diseases, such as Zika virus infection^22–25^. Thus, for JEV infection, brain organoids provide an ideal platform to study the pathogenesis and the antiviral reaction it induced.

In this study, we generated telencephalon organoids and infected these organoids with JEV. We hope to reveal what category of cells JEV prefer to infect in organoid, and how the JEV infection induces pathological alterations in organoid spheres. Finally, we are also interested in how the infected cells respond to the viral infection, particular cells at different stages of neural differentiation.

## Results

### Generation of telencephalon cortical organoids from hESCs

**Fig. 1:**
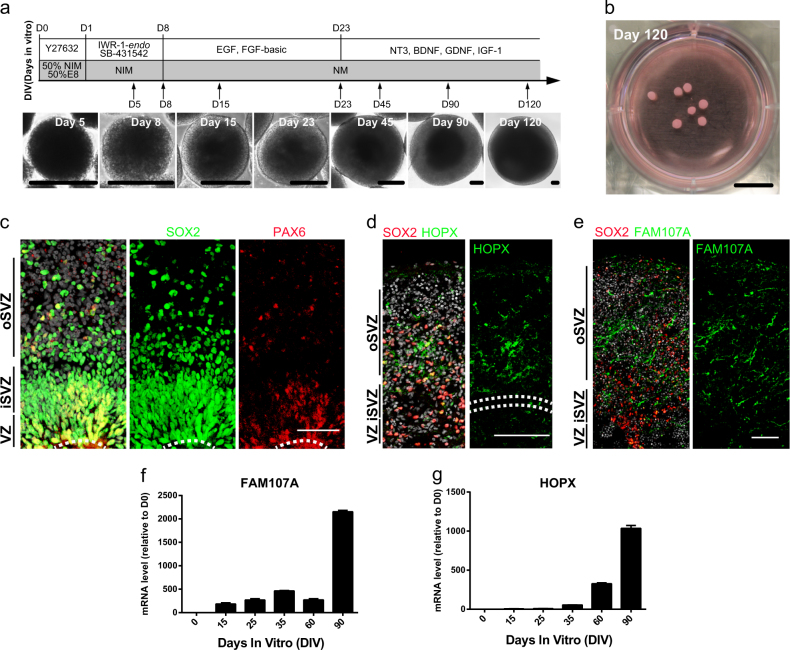
Generation of telencephalon cortical organoids from hESCs. **a** Schematic diagram of telencephalon cortical organoids derived from hESCs. Phase images of sample at different stages are shown. Scale bars: 200 μm. **b** Morphology of cortical organoids in a plate at day 120 in vitro. Scale bar: 1 cm. **c** Immunostaining of ventricular radial glia marker PAX6 (red) and neural progenitor cells marker SOX2 (green) in Hoechst-stained (HO, gray) telencephalon cortical organoids at D60, indicating the presence of a VZ-like and oSVZ-like region organized around a lumen (white dotted line). Scale bar: 50 μm. **d**, **e** Sample tiling images of immunostaining of oRGC markers HOPX (**d**) and FAM107A (**e**) in telencephalon cortical organoids. Scale bars: 100 μm. **f**, **g** Temporal pattern of oRGC markers *FAM107A* (**f**) and *HOPX* (**g**) expression in different stages of cortical organoids. Values represent mean ± SD, *n* = 3 for each point

We generate telencephalon cortical organoids from human embryonic stem cell (hESC) lines H9 (WA09) following a modified protocol^26^ (Fig. 1a). Telencephalon cortical organoids grow in suspension for long term, reach up to 2.5 mm in diameter after 120 days and remain viable thereafter (Fig. 1b). In cortical organoids of day 35, well-defined polarized neuroepithelial cells form structures resembling neural tubes. These structures are composed of nearly pure population of NESTIN^+^ SOX2^+^ neural progenitor cells (NPCs) that also express adherent junction markers β-CATENIN (Supplementary Fig. 1a). Inside the spheres near the lumen representing areas near the ventricular surface, ventricular radial glia (vRG) marker PAX6 and G2/M proliferation marker phosphohistone H3 (PH3) are expressed (Supplementary Fig. 1b), and the PAX6^+^ SOX2^+^ NPCs in these VZ-like structures are thought to be vRG cells (Fig. 1c). The VZ-like zone is surrounded by an intermediate region rich in TBR2^+^ cells resembling the subventricular zone (SVZ) (Supplementary Fig. 1c). Similarly, telencephalon cortical organoids derived from other hESC lines such as Q-CTS-hESC-1 (a clinical-grade hESC line)^27^ also exhibit multiple progenitor zones at day 45 (Supplementary Fig. 1d).

Human embryonic cerebral cortex possesses an expanded SVZ, which is further divided into the internal and outside parts named iSVZ and oSVZ, respectively. The latter is unique in primates for the outer radial glial cells (oRGCs) that produce most cortical neurons, by either multiple symmetrical or asymmetric divisions^28^. The oRG cells preferentially express genes associated with extracellular matrix formation and cell migration, such as TMEM14B, TNC, PTPRZ1, FAM107A, HOPX, and LIFR^29–31^. In the hESC-derived cortical organoids, HOPX^+^ SOX2^+^ oSVZ-like cells form a layer surrounding the VZ-like layer at day 55 of culture (Fig. 1d), similarly, another oRG marker FAM107A was also clearly expressed in oSVZ (Fig. 1e). mRNA of FAM107A and HOPX in cortical organoids at day 0, 15, 25, 35, 60, and 90 well correlates the stages of organoid development (Fig. 1f, g). Markers of all six neuronal subtypes are expressed in the organoids accordingly (Supplementary Fig. 1e–h), such as CUX1 and TBR1 of deep layer cortical neurons and REELIN of Cajal-Retzius neurons (Supplementary Fig. 1g). CTIP2, SATB2, and BRN2-expressing neurons are also detected (Supplementary Fig. 1h). All data indicate cortical organoids recapitulate the lamination of human fetal neocortex and form multi-layer progenitor zones including a prominent oSVZ layer that encompasses oRG progenitors.

Based on comparisons to published datasets of different human fetal organs^32^ and of human cortical sub-regions, Pearson’s correlation analysis show that organoids of days 90 and 190 well correlate to fetal brain and spinal cord, particularly the prefrontal cortex (PFC), but not other fetal somatic tissues (Supplementary Fig. 2a, b). Half of the cells fire single or multiple action potentials upon injection of depolarizing currents (Supplementary Fig. 2c, d, *n* = 14), elicit voltage-gated sodium and potassium currents (Supplementary Fig. 2e), fire spontaneous action potentials (Supplementary Fig. 2f), and exhibit membrane capacitance, membrane resistance (Supplementary Fig. 2g, h), and hyperpolarized resting membrane potentials around −50 mV (Supplementary Fig. 2i).

Together, these results demonstrate that our cortical organoids exhibit multi-layer progenitor zones and all six neuronal subtypes, which resemble human cortical development in vivo. In addition, the organoid development is similar to fetal human cortical development at the molecular level, as well as remaining neuronal electrophysiological activity.

### Modeling JEV infection with cortical organoids

**Fig. 2:**
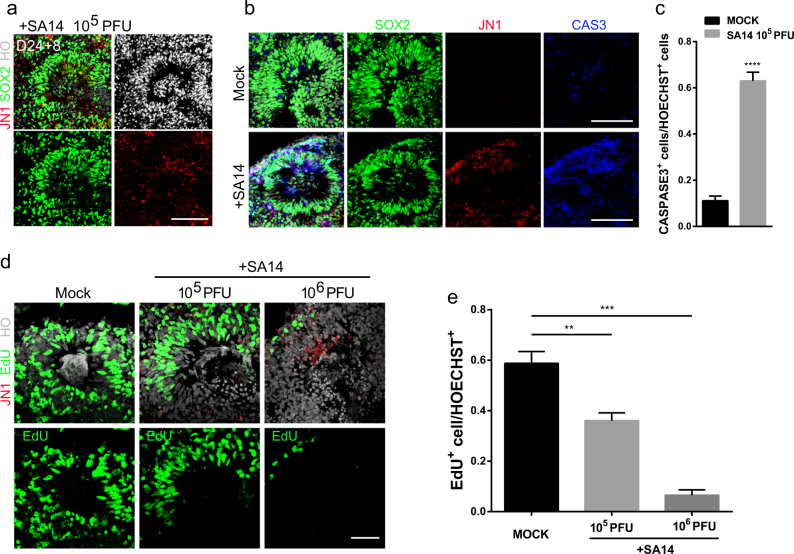
Modeling JEV infection during cortical neurogenesis. Brain organoids at different stages were inoculated with JEV (SA14, 10^5^ or 10^6^ PFU) or mock treated for 24 h and analyzed at day 8. **a** Sample immunostaining images of neural progenitor cells marker SOX2 (green) and JEV NS1 protein (JN1, red) in telencephalon cortical organoids exposed to JEV (SA14, 10^5^ PFU) or mock treated at day 24. Scale bar: 50 μm. **b** Immunostaining images of SOX2 (green), JN1 (red), and caspase-3 (blue) in telencephalon cortical organoids exposed to JEV (SA14, 10^5^ PFU) or mock treated. Scale bars: 50 μm. **c** Statistics for caspase3^+^ cells/HOECHST^+^ cells in cortical structures. Values represent mean ± SEM; *n* = 6, ****p < 0.0001. **d** EdU labeling of telencephalon cortical organoids exposed to JEV (10^5^ PFU), JEV (10^6^ PFU), or mock treated. EdU (green) treated for 24 h and analyzed at day 8, and JEV NS1 protein (JN1, red) was also immunostained. Scale bar: 50 μm. **e** Statistics for EdU^+^ cells/HOECHST^+^ cells. Values represent mean ± SEM, *n* = 5, **p < 0.01, ***p < 0.001

To establish an in vitro model of JEV infection, we induce human cortical organoids from H9-ESC line and infect them with JEV virulent strain SA14^33^ at different stages of cortical organoid culture. 8 days infection on cortical organoids of day 24, as well as day 9 and day 55, all demonstrate that JEV tend to infect SOX2^+^ hNPCs (Fig. 2a and Supplementary Fig. 3a, b). JEV infection causes overall smaller organoid and thinner neuronal layer, possibly by activating cell apoptosis (Fig. 2b, c). JEV also dose-dependently declines EdU^+^-proliferating cells (Fig. 2d, e). Together, cortical organoid allows for quantitative JEV exposure and infection, and then induces cell death and hNPC proliferation suppression.

### JEV infect hNPCs and oRGCs

To examine the JEV infection profile in different human neural cells, organoids are first dissociated into single cells and differentiated to hNPCs and immature neurons, respectively. After JEV (SA14) infection, a group of SOX2^+^ hNPCs are JN1^+^ (JEV NS1 glycoprotein) (Supplementary Fig. 4a). JN1^+^ TUJ1^+^ human immature neurons also exist (Supplementary Fig. 4c). These data indicate that hNPCs, as well as immature neurons, also support the viral growth and produce extracellular infectious JEV virions from 3 days post-infection in vitro (Supplementary Fig. 4b, d).

**Fig. 3:**
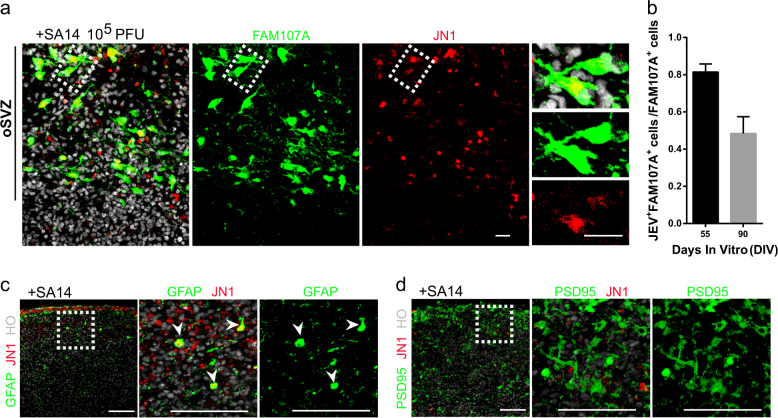
JEV targets oRGCs in cortical organoids during development. Brain organoids at different stages were inoculated with JEV (SA14, 10^5^ PFU) or mock treated for 24 h and analyzed at day 8. **a** Immunostaining of oRGC markers FAM107A (green) and JEV NS1 protein (JN1, red) in cortical organoids. White dashed box point to JN1^+^ FAM107A^+^ oRGC-like cells in the oSVZ region. Scale bars: 20 μm. **b** Statistics for JN1^+^ FAM107A^+^ cells/FAM107A^+^ cells in the oSVZ region demonstrates that JEV can targets oRGCs at days 55 and 90, respectively. Values represent mean ± SEM; *n* = 5. **c** Immunostaining of astrocyte marker GFAP (green) and JN1 (red) in Hoechst-stained (HO, gray) telencephalon cortical organoids at day 91. White arrows point to the existence of JN1^+^ GFAP^+^ astrocytes. Scale bars: 100 μm. **d** Immunostaining of mature neurons marker PSD95 (postsynaptic density-95 kDa, green) and JN1 (red) in cortical organoids at day 91. The results illustrate that mature neurons, which almost lack JN1 expression, generally are resistant to JEV infection. Scale bars: 100 μm

In cortical organoids of day 55, 81.42% of oRGCs (81.42 ± 1.96%, *n* = 5 organoids) in the oSVZ are infected with JEV. In that of day 90, 48.40% (48.40 ± 4.02%, *n* = 5 organoids) of oRGCs are FAM107A^+^ JN1^+^ (Fig. 3a, b). In cortical organoids older than day 100, comparable to PCW18, JEV prefers to infect GFAP^+^ astrocytes (Fig. 3c), instead of PSD95^+^ mature neurons (Fig. 3d). In summary, JEV prefers to infect younger NPCs, GFAP^+^ astrocytes, and oRGCs in oSVZ.

### Human cortical organoids gradually attain antiviral immunity response

**Fig. 4:**
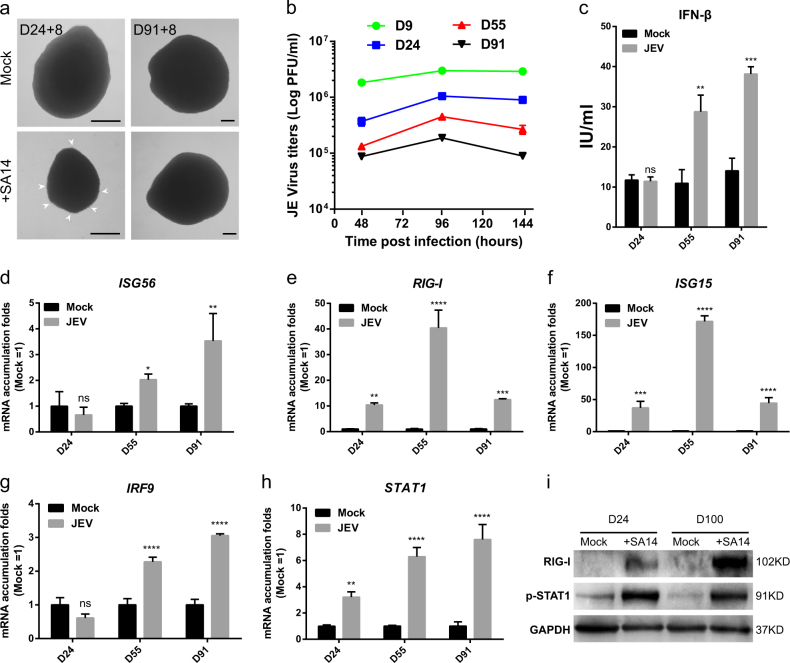
Innate antiviral immune response stimulated by JEV infection attained gradually during the development of cortical organoids. Brain cortical organoids at different developmental stages were inoculated with JEV (SA14, 10^5^ PFU) or mock treated for 24 h. **a** Bright-field microscopic images of organoids at day 24 (D24) and 91 (D91) are shown. Arrows point to detached cells. Scale bars: 200 μm. **b** JEV titers in supernatant of cultured organoids were determined by plaque-forming assay at different time post infection. **c** Interferon β in supernatant of cultured brain organoids was measured by ELISA. **d**–**h** Gene expression of *ISG56* (**d**), *RIG-I* (**e**), *ISG15* (**f**), *IRF9* (**g**), and *STAT2* (**h**) was quantified by qRT-PCR. **i** Western blots data showing the alterations of p-STAT1 and RIG-I in protein levels. Brain cortical organoids at day 24 and day 100 were inoculated with JEV (SA14, 10^5^ PFU) or mock treated for 24 h and analyzed by western blot with indicated antibodies on 2 days post infection

Infection of JEV generates distinct phenotypes in cortical organoids of day 24 versus day 91. The former usually exhibit much more severe outcomes with smaller spheres, irregular surrounding tissue and reduced surface (Fig. 4a). Accordingly, in older cortical organoids, virus titers in supernatants are also much lower (Fig. 4b). These data indicate that cortical organoids at early stages are more susceptive to JEV.

Innate immune response is important for host defense against viral infection during the early phase of infections^34^. JEV exposure does not cause IFN-β secretion in organoids of day 24 (Fig. 4c). However, organoids of long-term differentiation increase IFN-β protein level and induce the expression of ISGs such as *ISG56* (Fig. 4d) and *IFITM3* (Supplementary Fig. 5a), as well as *ISG54* and *OAS1* (Supplementary Fig. 5b, c).

More importantly, the expression level of RIG-I, one of the most important PRRs, is upregulated upon JEV infection in organoids of various stages (Fig. 4e, i), and *MDA5* is expressed at relatively low level (Supplementary Fig. 5d), indicating the different roles of RIG-I and MDA5 in responding to JEV infection in organoids. The results are in agreement with previously reported findings using transgenic mice model^35^. Toll-like receptor (TLR) genes like *TLR2*, *TLR3*, and *TLR7*, however, are not induced upon JEV infection (Supplementary Fig. 5e-g). The gene expression level of *IRF3* and *IRF7* was not changed upon JEV infection (Supplementary Fig. 5h, i). Interestingly, we found that *ISG15* is consistently expressed as RIG-I upon JEV infection in brain organoids (Fig. 4f). Our results indicate that host could recognize double-stranded RNA efficiently in organoids at its early development stage. IRF9 and phosphorylated-STAT1/2 could form the IFN-stimulated gene factor 3 (ISGF3) to induce expression of IFN-stimulated genes^12, 36, 37^. Interestingly, in JEV-infected organoids, IRF9 and p-STAT1 are increasingly expressed in more developed organoids upon JEV infection (Fig. 4g–i), which are in agreement with our results that the older cortical organoids have more effective antiviral activity (Fig. 4b).

## Discussion

Using hESC-derived cortical organoids, we revealed JEV tend to infect astrocytes and oRGCs of the developing human brain, inhibit cell proliferation, and induce cell death. Antiviral immunity of human brain is gradually established during development. These findings will help to understand the pathology of brain viral infection which in turn facilitate the development of effective therapeutics.

JEV infection causes irreversible brain damage, which remains a challenging issue across the world^38^. Because of the differential immune reactions between rodents and human, and lack of human brain tissues to study viral infection, very little is known about the pathology of JEV infection in human brain, and no cure is available for such kind of diseases. hESC-derived cortical organoids well recapitulate features of developing human brain cortex and are appropriate models to study a wide variety of brain diseases including viral infection. Here for the first time we establish a JEV infection model using hESC-derived cortical organoids.

Using this model, we identify JEV preferentially infect neural stem cells and oRGCs and cause brain developmental defects and microcepholon. In our model, there were apoptotic phenomena of CAS3^+^ in JEV-uninfected cells, at the same time, EdU^+^ cells were also observed in some cells infected by JEV, which is similar to previously reported in the organoids after ZIKV infection^21, 23^. We speculated that the decreased proliferation and increased apoptosis may be due to a secondary effect of virus-triggered inflammatory response. Early virus infection does not necessarily activate the cell apoptosis, so the expression of cell proliferation gene may not be directly inhibited. This interesting phenomenon has inspired us to explore how viral infections affect cell replication cycles.

**Fig. 5:**
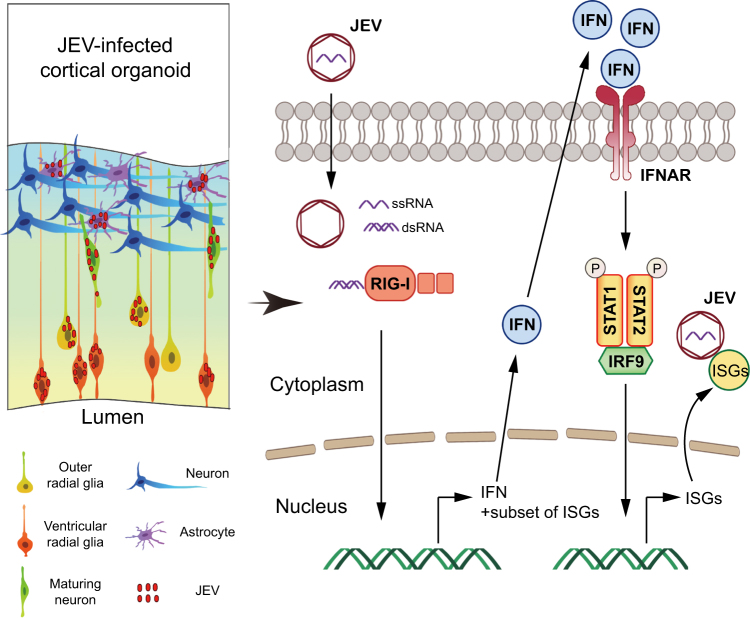
A bridged general view of JEV infection and its stimulated IFN signaling pathway in cortical organoid model. JEV infection impairs the development of organoids by targeting hNPCs, astrocytes, and outer radial glia cells. JEV was recognized by RIG-I after infection, and then ISG15 was independently associated with the expression of IFN-β. IRF9 cooperates with p-STAT1 to induce the expression of ISGs to inhibit JEV infection

JEV infection generates various outcomes from mild and transient symptoms to severely locomotive defects, depending on the age of infection, implying that in addition to the infection preference on brain cells, other aspects also affect the final outcomes of JEV infection. Innate immune response and other effect factors of the signaling pathway are also activated variously^39^. We reveal that JEV infection could upregulate the expression of RIG-I effectively, and may in turn induce the expression of IFN-β, following with activation of STAT1 and downstream ISGs expression. With the activation of IFN signaling pathway, IRF9 and STAT1 cooperate with each other to amplify the induction of late IFNs and ISGs genes. On the other hand, ISG15 is independently associated with the expression of IFN pathway, which is induced after JEV infection in organoids at the early developmental stage (Fig. 5). Consistently, recent studies also demonstrate that intrinsically expressed ISGs can protect stem cells against viral infection without activation of IFN signaling pathway^15^. It is possible that ISG15 has direct antiviral effect at different stage of development. In our system, p-STAT2 has not been detected to activate after JEV infection (Supplementary Fig. 5j, k). However, STAT2 may be involved in the formation of tri-complex or a homodimer, like unphosphorylated ISGF3 drives constitutive expression of ISGs to protect against viral infections^40–42^. Here, IFN-β was detected by ELISA only, because it has been reported that production and function of other type I and type III interferons are similar to that of IFN-β^43–45^. We also noted that it is very necessary to identify the differences between other type I or type III interferon and IFN-β in brain organoids in further studies.

In our system, organoids of day 24 encompass abundant NPCs. Certain genes in the upstream of IFN signaling pathway are temporarily inactive in such NPCs, cause refractory to IFN. Antiviral immunity is not only the host’s own defense mechanism, but also an important target for antiviral treatment. Our results show that IFN signaling pathway is not appropriate for defensing JEV infection at early developmental stage of brain cortical organoids, but provide a direction for searching future therapies. It is recently reported that mouse ESCs (mESCs) use an IFN-independent RNA interference-based mechanism for antiviral defense^46^, and RNAi is confirmed to work as an antiviral immunity in mammals^47^. Hence, there are more antiviral mechanisms that could be validated using our human brain organoids system.

## Materials and methods

### Maintenance of human embryonic stem cells cultures

hESC lines H9 (WA09) and clinical-grade hESC (Q-CTS-hESC-1) were cultured feeder-freely on Matrigel (BD Biosciences) coated 6-well plates in complete Essential 8 medium (GIBCO, Thermo Fisher Scientific). The colonies were manually passaged with 0.5 mM EDTA (Invitrogen; pH = 8) every 4–6 days and maintained at 37 °C with 5% CO_2_. All stem cell lines used in this study were regularly tested and maintained mycoplasma-free and with verified normal karyotype.

### Culture of human telencephalon cortical organoids

To generate suspended cellular aggregates of pluripotent cells, hESCs were cultured feeder-free on Matrigel-coated surface with Essential 8 medium. In brief, hESCs were exposed to a low concentration of dispase for 5–8 min. Suspended colonies were subsequently transferred into ultra-low-attachment plates, within medium containing 50% NIM (DMEM/F12, N2 supplement (Invitrogen; 100×), non-essential amino acids (Invitrogen; 100×), GlutaMAX (Invitrogen; 100×), Heparin (Sigma; 2 µg/ml)) and 50% Essential 8 medium. For the first 24 h (day 0), the medium was supplemented with 10 μM ROCK inhibitor Y-27632 (Merck). To reduce tissue heterogeneity and pre-pattern organoids towards the dorsal telencephalon fate, we pre-patterned embryoid bodies to the fate of a specific brain region. IWR-1-*endo* (Merck, 5 µM) and SB-431542 (Merck, 5 µM) were added to the NIM medium in the first 6 days (day 1–day 7). On day 8 of the protocol, floating neurospheroids were transferred to neural medium (NM) containing Neurobasal, B-27 supplement (Invitrogen; 50×) and GlutaMAX, which was supplemented with 10 ng/ml FGF-basic (Peprotech) and 20 ng/ml EGF (Peprotech) with every other day medium change (day 8–day 22). To promote neural progenitors to differentiate to neurons, organoids subsequently cultured in NM with 10 ng/ml BDNF (Peprotech), 10 ng/ml GDNF (Peprotech), 10 ng/ml IGF-1 (Peprotech), and 20 ng/ml NT3 (Peprotech) starting at day 23. Medium changes every 3 or 4 days. From beginning of differentiation culture, all EBs and organoids were maintained at 37 °C with 5% CO_2_.

### Histology and immunofluorescence

Cells and organoids were all fixed with 4% (w/v) paraformaldehyde (Sigma) and 4% sucrose in phosphate-buffered saline (PBS). Organoids were then incubated in 30% (w/v) sucrose solution overnight at 4 °C. Next, organoids were placed in tissue base molds and embedded within O.C.T. compound (Tissue-Tek, Hatfield, PA) at −20 °C. Organoids blocks were then stored at −80 °C or used for cryosectioning to obtain 20 μm slices using freezing microtome (Leica). The cryosections were washed with washing buffer (1× PBS, 0.3% Triton-100) for three times (5 min for each time) at room temperature (RT), then fixed with 4% paraformaldehyde in PBS for 15 min and blocked in PBS buffer containing 10% donkey serum and 0.3% Triton X-100 (Sigma) for 1 h at RT, followed by the incubation with the primary antibodies at 4 °C overnight with 5% donkey serum and 0.15% Triton X-100. The cryosections were incubated with secondary antibodies containing 5% donkey serum and 0.15% Triton X-100 for 1 h. Nuclei were counterstained with Hoechst 33342 DNA dye (Life Technologies, 1: 1000) at RT for 10 min and mounted on glass slides. Images were taken on a Carl Zeiss LSM710 confocal microscope and processed using ZEN 2012 software. The following primary antibodies were used for immunofluorescence: PAX6 (Santa Cruz Biotechnology; mouse, 1:100), human-SOX2 (R&D; goat, 1:1000), TBR2 (Abcam; rabbit, 1:500), Phospho-Histone H3 (Cell Signaling Tec; rabbit, 1:500), CTIP2 (Abcam; rat, 1:500), BRN2 (Santa Cruz; goat, 1:200), TBR1 (Abcam; rabbit, 1:200), SATB2 (Abcam; rabbit, 1:100), CUX1 (Santa Cruz; rabbit, 1:200), REELIN (Millipore; mouse, 1:300), FAM107A (Sigma; rabbit, 1:200), HOPX (Santa Cruz; rabbit, 1:200), JEV NS1 glycoprotein, JN1 (Abcam; mouse, 1:20), Cleaved Caspase-3 (Cell Signaling Tec; rabbit, 1:1000).

### Organoid slice preparation for electrophysiology

Organoid slices were prepared by embedding organoids in 4% low melting point agarose cooled to approximately 32 °C. Slices (250 μm) were sectioned using a vibratome (7000 smz 2, Campden Instruments, Loughborough, UK) in ice-cold cutting ACSF, and then stored at RT in artificial cerebral spinal fluid, containing: NaCl 125 mM, KCl 2.5 mM, MgCl_2_ 1 mM, NaH_2_PO_4_ 1.25 mM, CaCl_2_ 2 mM, NaHCO_3_ 25 mM, D-glucose 25 mM (290–310 mosm/kg, pH 7.4). ACSF was oxygenated (95% O_2_, 5% CO_2_). Slices were let recover for at least 60 min prior to electrophysiological recordings. All chemicals were obtained from Sigma.

### Patch-clamp recordings

Whole-cell current-clamp recordings were performed at 22 °C in artificial cerebral spinal fluid, bubbled with 95% O_2_ and 5% CO_2_. Borosilicate glass electrodes (resistance 6–10 MΩ) were filled with an intracellular solution containing 135 mM potassium gluconate, 7 mM NaCl, 10 mM HEPES, 2 mM MgATP, 0.3 mM Na_2_GTP, and 2 mM MgCl_2_, adjusted to pH 7.4 with KOH. Cell visualization and patch pipette micromanipulation were performed by video microscopy, employing a 40× water-immersion objective mounted on an upright microscope equipped with infrared differential interference contrast (Nikon, Eclipse fn1, Japan). Intracellular membrane electrical potentials were recorded in current-clamp mode, using a Multiclamp 700B amplifier (Molecular Devices, Palo Alto, CA, USA). Data were digitized at 10 kHz with a 2 kHz low-pass filter. Data were analyzed using Clampfit 10.6 (Axon Instruments). For voltage-clamp recordings, cells were held at −70 mV.

### Cell lines and viruses

BHK-21 cells were cultured in DMEM (Thermo Fisher Scientific) supplemented with 10% FBS and 1% P.S. at 37 °C in 5% CO_2_. C6/36 cells were cultured in RPMI 1640 (Thermo Fisher Scientific) supplemented with 10% FBS at 30 °C in 5% CO_2_. Strain SA14 was from the Chinese National Institute for Food and Drug Safety and it was propagated in C6/36 cells cultured in RPMI 1640 with free FBS. Virus stocks were stored in aliquots at −80 °C. Virus titers were determined by plaque-forming assay in BHK-21 cells. Briefly, BHK-21 cells were seeded in a 12-well plate for 24 h, and then cells were infected with diluted viruses for 1 h. Viral supernatant was replaced with DMEM containing 1% low melting agarose and 1% FBS. Viral plaques were developed at 3 d.p.i. (day post-infection).

### Interferon β detection by ELISA

Interferon β in supernatant of cultured brain organoids was measured by an ELISA Kit (PBL Assay Science). The experiment was performed according to the kit’s manual. Standard samples for the generation of a standard curve were also provided in the kit. Samples were diluted to the range of quantification of the kit using the dilution buffer. Optical density (OD) was measured at 450 nm with a microplate reader (Backman).

### Western blotting

Samples were lysed using by RIPA (Thermo Fisher Scientific) containing protease inhibitor (Roche). Protein were quantified and 10 μg of each lysate were loaded per lane of a NuPAGE™ 4–12% Bis–Tris Protein Gel (Thermo Fisher Scientific). Samples were separated on 200 V for 45 min. Then protein samples were then transferred to Puro Nitrocellulose Blotting Membranes (PALL) on 200 mA for 2 h. The membrane was blocked in 3% BSA in Tris-based saline with Tween 20 (0.1% TBST) buffer for 1 h and followed by incubating with primary antibodies overnight at 4 °C. Next day, membranes were washed three times and then incubated with HRP-conjugated secondary antibodies for 1.5 h at RT. Protein bands were visualized using SuperSigna West Pico PLUS Chemiluminescent Substrate (Thermo Fisher Scientific) and blot images were captured by Automatic chemiluminescence image analysis system (Tanon). The dilution of antibodies used in Western blotting is as followed: phospho-STAT1 (Ser727) (CST, #8826), phospho-STAT2 (Tyr690) (CST, #4441), RIG-I (CST, #4200), anti-GAPDH-ChIP Grade (Abcam, ab9485) 1:1000.

### Genome quantification by SYBR green qRT-PCR

Total RNAs were isolated from brain organoids using Trizol (Qiagen). Gene expression levels were quantified by one-step SYBR green qRT-PCR (TAKARA), normalized against *GAPDH*. The primers used in this paper and primer sequence for qRT-PCR are shown below.

**Table:** 

***IRF*** **-3-F**	**AGAGGCTCGTGATGGTCAAG**
***IRF*** **-3-R**	**AGGTCCACAGTATTCTCCAGG**
***IRF*** **-7-F**	**GCTGGACGTGACCATCATGTA**
***IRF*** **-7-R**	**GGGCCGTATAGGAACGTGC**
***ISG54*** **-F**	**AAGCACCTCAAAGGGCAAAAC**
***ISG54*** **-R**	**TCGGCCCATGTGATAGTAGAC**
***ISG56*** **-F**	**TTGATGACGATGAAATGCCTGA**
***ISG56*** **-R**	**CAGGTCACCAGACTCCTCAC**
***OAS1*** **-F**	**CTGACFCTGACCTGGTTGTCT**
***OAS1*** **-R**	**CCCCGGCGATTTAACTGAT**
***IFITM3*** **-F**	**CATCCTCATGACCATTCTGC**
***IFITM3*** **-R**	**TCAGTGATGCCTCCTGATCT**
***STAT1*** **-F**	**CAGCTTGACTCAAAATTCCTGGA**
***STAT1*** **-R**	**TGAAGATTACGCTTGCTTTTCCT**
***STAT2*** **-F**	**CCAGCTTTACTCGCACAGC**
***STAT2*** **-R**	**AGCCTTGGAATCATCACTCCC**
***IRF9*** **-F**	**GCCCTACAAGGTGTATCAGTTG**
***IRF9*** **-R**	**TGCTGTCGCTTTGATGGTACT**
***RIGI*** **-F**	**CTGGACCCTACCTACATCCTG**
***RIGI*** **-R**	**GGCATCCAAAAAGCCACGG**
***ISG15*** **-F**	**GAGAGGCAGCGAACTCATCT**
***ISG15*** **-R**	**CTTCAGCTCTGACACCGACA**
***FAM107A*** **-F**	**GCAGCGTGTCCTAGAGCAC**
***FAM107A*** **-R**	**CCGCAGGTTTTCCCTGACT**
***HOPX*** **-F**	**GAGACCCAGGGTAGTGATTTGA**
***HOPX*** **-R**	**AAAAGTAATCGAAAGCCAAGCAC**

### Quantification and statistical analysis

Statistical analyses were performed using Prism 6 software (GraphPad Prism). For all experiments with error bars, data were presented as the mean ± SEM or ± SD. The unpaired two-tailed Student’s *t* test was used to calculate statistical significance between two groups with *p* values. Comparisons among three groups or more, statistical significance were made using two-way ANOVA analyses. A value of *p* < 0.05 was considered to be significant. *p < 0.05, **p < 0.01, ***p < 0.001, ****p < 0.0001.

## Electronic supplementary material

Supplemental Information

Supplemental Fig. 1

Supplemental Fig. 2

Supplemental Fig. 3

Supplemental Fig. 4

Supplemental Fig. 5
