# Supplementary material for: Differential antiviral immunity to Japanese encephalitis virus in developing cortical organoids
Source: Cell Death Dis. 2018 Jun 18;9(7):719. doi: 10.1038/s41419-018-0763-y (PMC6006338; doi:10.1038/s41419-018-0763-y)
Supplement: Supplementary file 1 — Supplemental Information [file 41419_2018_763_MOESM1_ESM.pdf]

Supplementary Information

The Supplementary Information file contains Supplementary Fig. 1 (Cortical organoids contain multi-layer progenitor zones and six cortical layers); Supplementary Fig. 2 (Global transcriptome analysis and electrophysiological properties of developing cortical organoids); Supplementary Fig. 3 (Modeling JEV infection using cortical organoids); Supplementary Fig. 4 (JEV can targets hESC-derived cortical NPCs and immature neurons); Supplementary Fig. 5 (Gene expression of *ISG*s, *TLR*s and *IRF*s in brain organoids upon JEV infection).

Supplementary Figure Legends

Supplementary Fig. 1. Cortical organoids contain multi-layer progenitor zones and six cortical layers. **a** Immunohistochemistry of SOX2 (green), β-CATENIN (red) and NESTIN (blue) at day 35 cortical organoid, demonstrate the presence of a neuroepithelium-like structures near a lumen (white dotted line). Scale bar: 50μm. **b** Immunostaining for $PH3^+$ (green) $PAX6^+$ (red) proliferating cells in a Hoechst-stained (HO, gray) cortical organoid. Scale bar: 50μm. **c** Intermediate progenitor cells marker TBR2 (red) are present in a SVZ-like region beyond the $PAX6^+$ (green) VZ-like region. Scale bar: 20μm. **d** Q-CTS-hESC-1-derived cortical organoids contained $SOX2^+$ (green) $PAX6^+$ (red) and $TBR2^+$ (blue) multi-layer progenitor zones. Scale bar: 50μm. **e** Cortical plate marker CTIP2 (green) at day 70 organoids. Scale bar: 100μm. **f** Schematic representation of marker expression for all six cortical layers. **g** Immunostaining for deep cortical layers marker CUX1 (green), TBR1 (red), and Cajal-Retzius neuron cell marker REELIN (blue) at day 120 cortical organoid. Scale bar: 50μm. **h** Immunohistochemical analyses demonstrate the presence of early-born $CTIP2^+$ (green) layer, late-born $SATB2^+$ (red) neurons and $BRN2^+$ (blue) neurons at day 120 cortical organoid. Scale bar: 50μm.

Supplementary Fig. 2. Global transcriptome analysis and electrophysiological properties of developing cortical organoids. a-b Heatmap of Pearson's correlation analysis of RNA-seq datasets at day 90 and 190 cortical organoids, with Roost et al. published datasets from 21 different human fetal organs (**a**) and Allen Brain Atlas's published datasets from 3 different cortical subregions (**b**). **c** An example of cells under the patch-clamp in a day 300 cortical organoid. **d** Representative traces of membrane potential responding to step depolarization by current injection steps from −10 pA to +30 pA in 10-pA increments. Membrane potential was current-clamped at around -70 mV. **e** Representative traces of whole-cell currents in voltage-clamp mode; cells were held at -70 mV; step depolarization from -80mV to +60 mV at 10-mV intervals was delivered. The inset shows Na^+ currents. **f** Spontaneous action potentials recorded from cell of cortical organoid slice. No current injection was applied. **g-i** Quantification of resting membrane potential (RMP, **g**), membrane capacitance (*Cm*, **h**) and membrane resistance (*Rin*, **i**). n=7 cortical organoid neurons.

Supplementary Fig. 3. Modeling JEV infection using cortical organoids. a-b Immunostaining images of SOX2 (green), JN1 (red) and caspase-3 (blue) in Hoechst-stained (HO, gray) cortical organoids exposed to JEV (SA14, 10^5PFU) or mock treated at day 9 (**a**) and day 55 (**b**). While arrows point to detached cells. Scale bars: 50μm.

Supplementary Fig. 4. JEV can targets hESC-derived cortical NPCs and immature neurons. a-b Cortical neural progenitor cells exposed to JEV (SA14, 10^5 PFU) or mock treated for 24 hrs and analyzed 24 hrs later. Immunostaining images (**a**) and JEV titers (**b**) were shown. Scale bar: 100μm. **c-d** Cortical immature neurons exposed to JEV (SA14, 10^5 PFU) or mock treated for 24 hrs and analyzed 2 days later. Immunostaining images (**c**) and JEV titers (**d**) were shown. Scale bar: 100μm.

Supplementary Fig. 5. Gene expression of *ISG*s, *TLR*s and *IRF*s in brain organoids upon JEV infection. a–j Gene expression of *IFITM3* (**a**), *ISG54* (**b**) *OAS1* (**c**), *MDA5* (**d**), *TLR2* (**e**), *TLR3* (**f**), *TLR7* (**g**), *IRF3* (**h**), *IRF7*(**i**) and *STAT2* (**j**) was quantified by qRT-PCR. **k** Western blots data showing the alterations of p-STAT2 in protein levels. Brain cortical organoids at day 24 and day 100 were inoculated with JEV (SA14, 10^5 PFU) or mock treated for 24 hours and analyzed by western blot with indicated antibodies on 2 days post infection.
