# Supplementary figures and images for: Differential antiviral immunity to Japanese encephalitis virus in developing cortical organoids
Source: Cell Death Dis. 2018 Jun 18;9(7):719. doi: 10.1038/s41419-018-0763-y (PMC6006338; doi:10.1038/s41419-018-0763-y)

Supplemental Fig.1

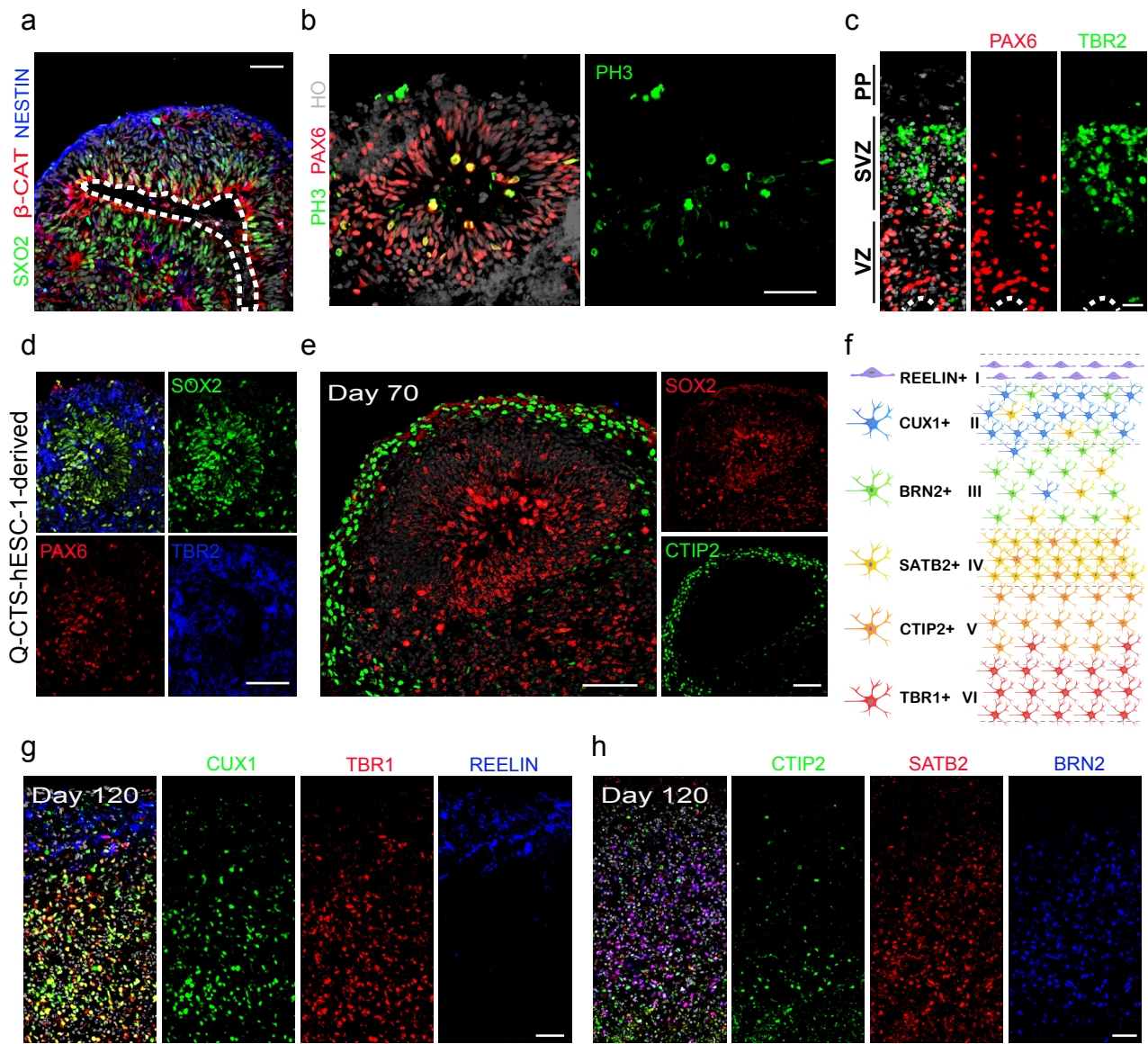

Supplement: Supplementary file 2 — Supplemental Fig. 1 [file 41419_2018_763_MOESM2_ESM.pdf]

Supplemental Fig.2

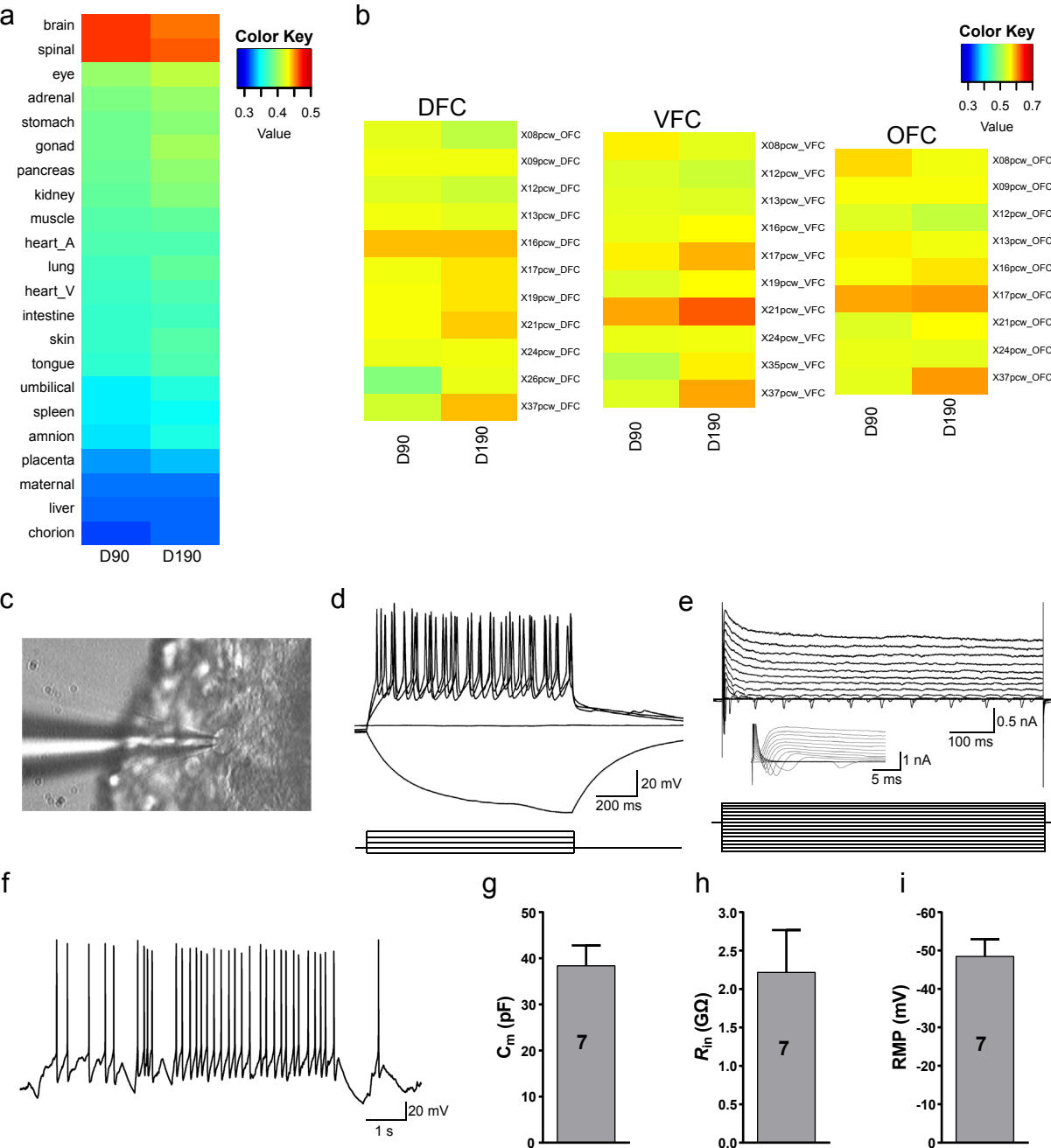

Supplement: Supplementary file 3 — Supplemental Fig. 2 [file 41419_2018_763_MOESM3_ESM.pdf]

Supplemental Fig.3

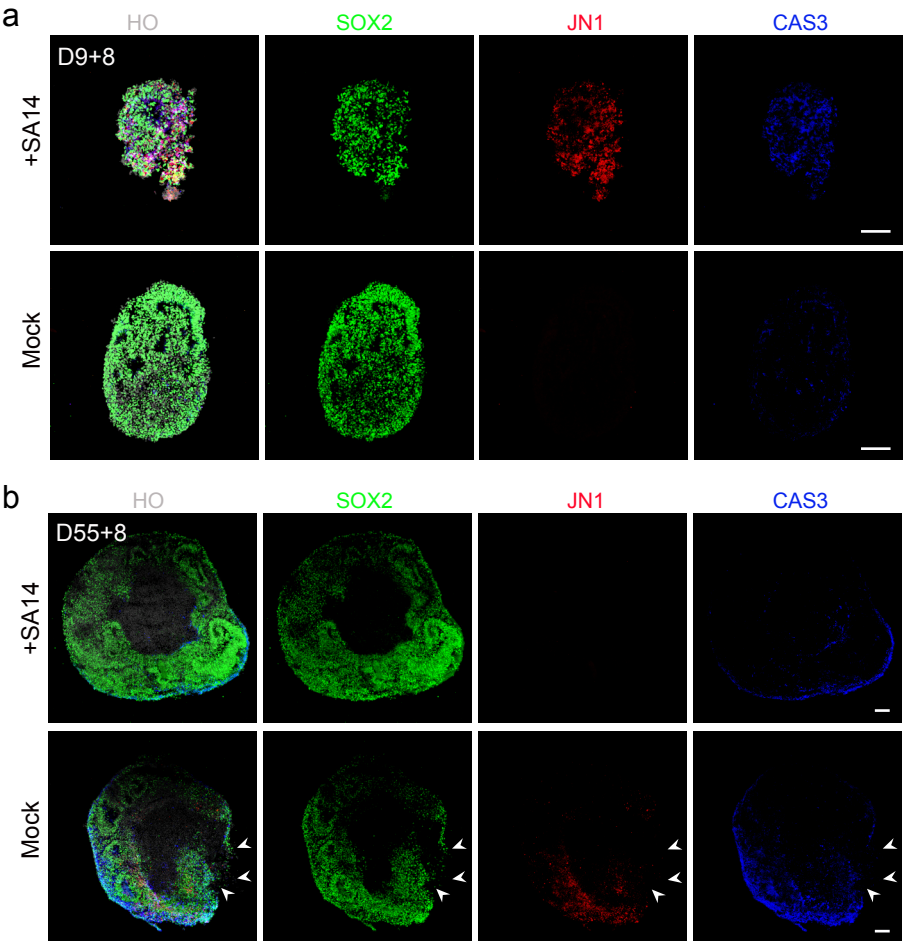

Supplement: Supplementary file 4 — Supplemental Fig. 3 [file 41419_2018_763_MOESM4_ESM.pdf]

Supplemental Fig.4

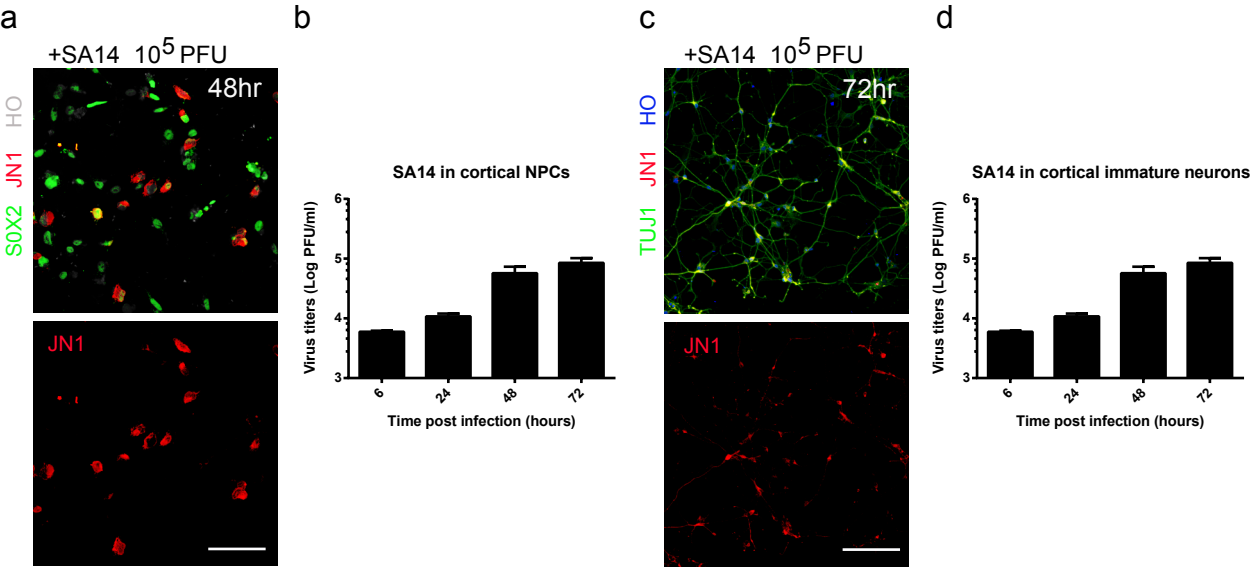

Supplement: Supplementary file 5 — Supplemental Fig. 4 [file 41419_2018_763_MOESM5_ESM.pdf]

Supplemental Fig.5

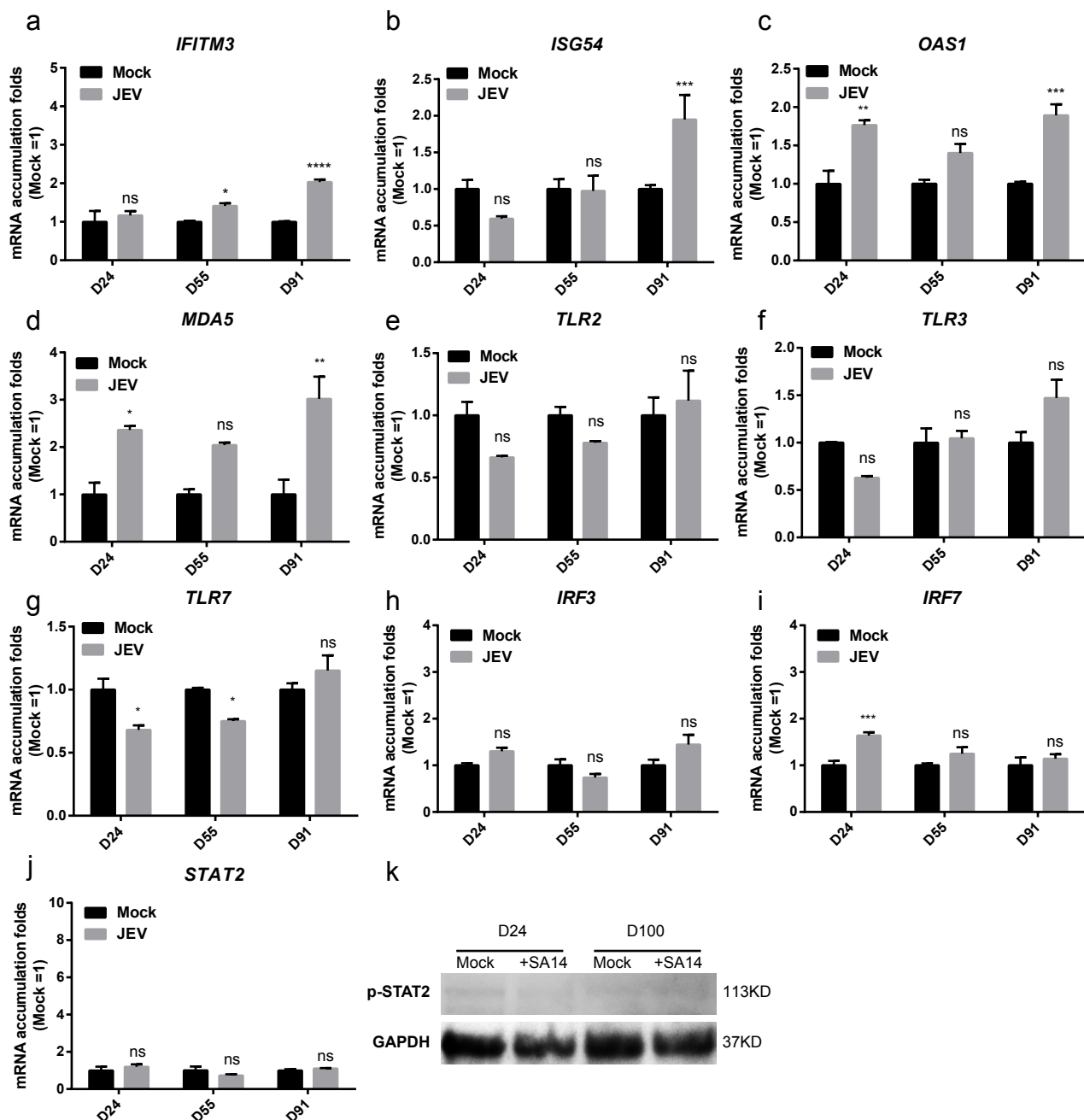

Supplement: Supplementary file 6 — Supplemental Fig. 5 [file 41419_2018_763_MOESM6_ESM.pdf]
